# The role of deep eutectic solvents and carrageenan in synthesizing biocompatible anisotropic metal nanoparticles

**DOI:** 10.3762/bjnano.12.69

**Published:** 2021-08-18

**Authors:** Nabojit Das, Akash Kumar, Raja Gopal Rayavarapu

**Affiliations:** 1 Nanomaterial Toxicology Laboratory, Nanomaterial Toxicology Group, CSIR-Indian Institute of Toxicology Research (CSIR-IITR), Vishvigyan Bhawan, 31 Mahatma Gandhi Marg, Lucknow 226001, India; 2 Academy of Scientific and Innovative Research (AcSIR), Ghaziabad 201002, India

**Keywords:** anisotropic nanoparticles, carrageenan, cytotoxicity, eutectic solvents, surfactants

## Abstract

Plasmonic metal nanoparticles are widely used for many applications due to their unique optical and chemical properties. Over the past decade, anisotropic metal nanoparticles have been explored for imaging, sensing, and diagnostic applications. The variations and flexibility of tuning the size and shape of the metal nanoparticles at the nanoscale made them promising candidates for biomedical applications such as therapeutics, diagnostics, and drug delivery. However, safety and risk assessment of the nanomaterials for clinical purposes are yet to be made owing to their cytotoxicity. The toxicity concern is primarily due to the conventional synthesis route that involves surfactants as a structure-directing agent and as a capping agent for nanoparticles. Wet chemical methods employ toxic auxiliary chemicals. However, the approach yields monodispersed nanoparticles, an essential criterion for their intended application and a limitation of the green synthesis of nanoparticles using plant extracts. Several biocompatible counterparts such as polymers, lipids, and chitosan-based nanoparticles have been successfully used in the synthesis of safe nanomaterials, but there were issues regarding reproducibility and yield. Enzymatic degradation was one of the factors responsible for limiting the efficacy. Hence, it is necessary to develop a safer and nontoxic route towards synthesizing biocompatible nanomaterials while retaining morphology, high yield, and monodispersity. In this regard, deep eutectic solvents (DESs) and carrageenan as capping agent for nanoparticles can ensure the safety. Carrageenan has the potential to act as antibacterial and antiviral agent, and adds enhanced stability to the nanoparticles. This leads to a multidimensional approach for utilizing safe nanomaterials for advanced biomedical and clinical applications.

## Review

### Introduction

Plasmonic metals such as gold and silver, upon achieving nanoscale dimensions, exhibit unusual physicochemical characteristics, such as interesting plasmonic, optical and catalytic properties, and facile surface modification with tunable size and morphology [1]. Among these properties, the ability of surface plasmon resonance (SPR) at visible to near-infrared (NIR) wavelengths is the most striking characteristic feature of gold and silver nanoparticles. Surface plasmon resonance is an inherent property of plasmonic metal nanoparticles that is immensely employed as a tool for theranostics and is highly influenced by the size and shape of the nanoparticle [2]. The property of SPR has also been exploited for nanochips and smartphone-based sensing applications [3–5]. Several other advanced sensing applications have emerged, such as battery-free and wireless devices, providing on-site results [6–7]. NIR absorption is exclusively exhibited by plasmonic anisotropic nanoparticles, enabling diagnostic imaging within the optical therapeutic window. The realization of immense potential due to innate striking features of anisotropic nanoparticles has brought material and biological researchers under the same umbrella. The manifestation of NIR absorption in theranostic application is highly acknowledged due to the ability of NIR/IR rays to deeply penetrate tissues, enabling nanoparticle-mediated photothermal or contrast effects. However, the final purpose of these nanomaterials for biological applications is determined after successful toxicity assessment and stability evaluation in biological media [8]. Traditionally used stabilizing agents, such as surfactants and citrate, enable the synthesis of nanoparticles with high yield and monodispersity but also cause cytotoxicity and genotoxicity even at low concentrations [9–10]. Surfactants are known to act as a template for anisotropy in plasmonic metal nanoparticles, especially rod-shaped gold nanoparticles. The most approved and widely used surfactants for synthesizing anisotropic nanoparticles are quaternary ammonium surfactants with halides (bromide, chloride, or iodide) as counterions. Hexadecyltrimethylammonium bromide (CTAB) is the most commonly used surfactant for synthesizing anisotropic nanoparticles with high yield and monodispersity. The surfactant induces anisotropy during the growth of nanoparticles and enables NIR absorption capability due to longitudinal surface plasmon resonance (LSPR) [11]. However, despite the superior plasmonic properties, these nanomaterials are far away from a substantial use in biological applications due to toxic capping agents employed during synthesis.

Several counterparts such as polymers, lipids, and chitosan-based nanoparticles are extensively explored in drug delivery and therapeutic applications due to their biocompatible nature. Green synthesis of metal nanoparticles for biomedical applications has gained momentum recently due to their inherent nontoxicity. Although they are biocompatible, these metal nanoparticles lack monodispersity, high yield, and controlled morphology, which are essential criteria for the successful use in biological milieus. Recent studies indicated that the green synthesis of nanoparticles, such as zinc oxide nanoparticles and bimetallic copper–silver and nickel–cobalt nanoparticles, is preferred for catalytic, antibacterial, and therapeutic applications [12–14]. Several other synthesis methods have been developed for environmental applications, such as biohydrogen production and chromium deionization [15–18]. In addition, polymer-based nanoparticles showed low drug loading and encapsulation efficiency. The acidic nature of poly(lactic-*co*-glycolic acid) is not suitable for certain drugs and bioactive molecules and make the polymer prone to a higher enzymatic degradation rate [19]. This is why there is a need for novel synthetic routes for synthesizing safe plasmonic metal nanoparticles maintaining high yield and monodispersity with tunable size and morphology. Nontoxic, biocompatible, and sustainable solvents, such as deep eutectic solvents (DESs), and carrageenan as capping and reducing agent are gaining popularity in nanomaterial synthesis. Apart from potential tools for biomedical applications, recent studies have also shown the utilization of anisotropic nanomaterials in CO_2_ mitigation and climate change control [20–22]. Several other studies reported novel environmental remediation approaches based on nanomaterials [23–24].

Deep eutectic solvents (DESs) are a class of nascent sustainable, non-aqueous solvents, comparable to room-temperature ionic liquids (RTILs). DESs fairly resemble the RTILs even though there are important differences regarding ecological footprint and price. One is that DESs are predominantly composed of molecules unlike RTILs, which predominantly contain ions. Also, DESs are fairly cheaper and easier to prepare, do not generate waste during preparation, and require no further purification, which gives them properties of a green solvent [25]. However, DESs share remarkable features with RTILs such as low vapor pressure, high tolerance to humidity, and high thermostability. The term “deep eutectic solvent” was first coined by Abbott in the year 2003 [26]. The first work on using DESs as a solvent in synthesizing anisotropic gold nanoparticles was reported in 2008 [27]. The synthesis involved no surfactant or seed in the reaction mixture. Later on, several studies were carried out to synthesize nanomaterials using DESs that embrace the principles of green chemistry. Despite extensive studies for more than a decade, DESs as solvents for nanomaterial synthesis yet awaits exploration regarding biological applications. In an interesting recently published work, a natural deep eutectic solvent (NADES) has been used to extract metal oxide nanoparticles [28]. Numerous indispensable parameters including surface tension, polarity, viscosity, and hydrogen bonding have an important influence on the reactivity of species. Also, the formation of nanostructures is governed by the mass transport properties of the DES components. It is also possible to modulate the viscosity of DESs, especially NADES, by varying the composition ratio of hydrogen bond donor and hydrogen bond acceptor components [29]. Also, the growth mechanisms and nucleation processes of nanoparticles are highly modulated by the components of DESs through modifying reduction potentials, neutralizing charge, and in particular, crystal face pacification, enabling preferential crystal growth. DESs are the medium where nanoparticle synthesis occurs in the presence of capping agent and reducing agents. Biocompatible capping and reducing agents, such as carbohydrates (i.e., carrageenan), are suitable for nanoparticle synthesis intended for biological applications.

**Figure 1:**
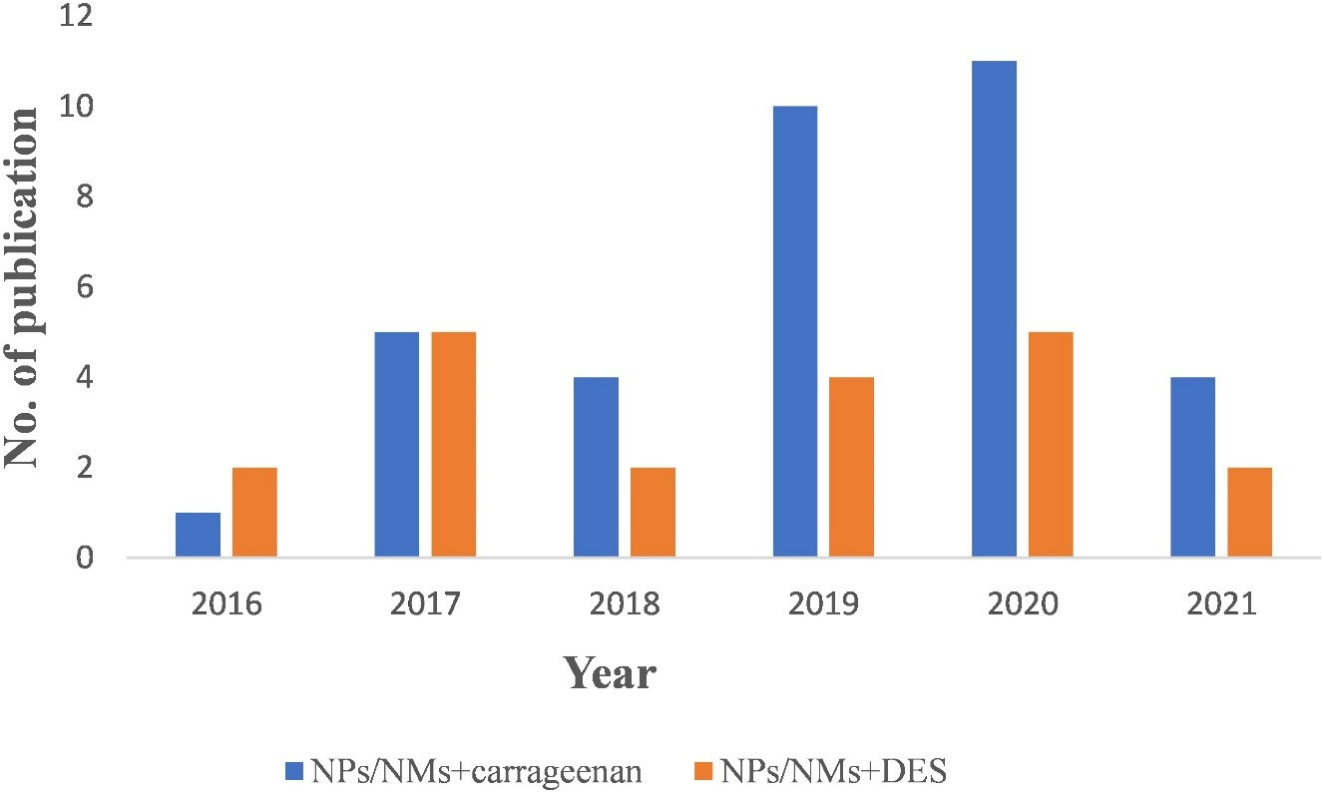
Number of publications over the last five years on the synthesis of nanoparticles (NPs)/nanomaterials (NMs) using carrageenan and a deep eutectic solvent (DES). The numbers have been obtained from SCOPUS-indexed journals using PubMed.

Carrageenans are a group of oligosaccharides predominantly found in Rhodophyceae commonly known as red algae. They are sulfated linear oligosaccharides consisting of ᴅ-galactose residue units linked by (1→3)-linked β-ᴅ-galactopyranose (unit G) and (1→4)-linked α-ᴅ-galactopyranose (unit D) alternatively. Some of the reports in the literature showed that carrageenan has several pharmacological properties such as antiviral and antitumor activity that can add pharmaceutical value to the nanomaterials synthesized using them [30–31]. These additional properties enabled carrageenan to emerge as a suitable alternative for other biocompatible molecules and biopolymers. Similar to DESs, carrageenan is also biocompatible. A good understanding of its role as a green component for synthesizing nanomaterial for biological applications is only at the beginning. To be more specific, carrageenan was used in the synthesis of plasmonic metal nanomaterials much later than DESs. The excellent properties of carrageenan as a stabilizing/capping and a reducing agent was reported in a recent study for gold nanoparticles [32]. To understand the applicability of DESs and carrageenan in nanotechnology, a histogram with the number of publications (Scopus-indexed journals) for the past five years in shown in Figure 1. This shows the emerging potential of these novel materials for nanobiotechnology research.

The histogram shows an upward trend regarding the use of carrageenan in nanobiotechnology, indicating that it is a safe approach in synthesizing biocompatible nanomaterials. Carrageenans were either used in synthesis as a capping agent or as functional molecule for nanoparticle stabilization and targeted drug delivery. In contrast, nanomaterials synthesized using DESs received less interest, which is evident through a stagnant number of reports over the last five years. However, twenty reports have been published on using DESs for nanomaterial synthesis.

This review attempts to illuminate the works from the last decade that involved DESs and carrageenan in the synthesis of nanomaterials that are nontoxic. The review begins by discussing widely used wet chemical methods of synthesizing anisotropic plasmonic metal nanomaterials. We also give insight in growth mechanisms during the initiation of anisotropy in the presence of a surfactant. This review is a crisp overview of determining the anisotropy–cytotoxicity relationship due to structure-directing agents and the role of DESs and carrageenan in alleviating toxicity of the synthesized nanomaterials. We conclude with an outlook towards the possible amalgamation of DESs and carrageenan creating a nontoxic platform for synthesizing nanomaterials with the potential for biological applications.

### Wet chemical reduction method using surfactants: pros and cons

The widely used wet chemical approach for synthesizing nanomaterials is a facile reduction method involving a precursor metal salt and a reducing agent in the dispersion phase [33]. Furthermore, a stabilizing/capping agent is used for enhanced stability and functionalization for the intended application. The wet chemical route allows for a high degree of controllability and reproducibility in synthesizing anisotropic nanomaterials maintaining high yield and monodispersity. Initially, gold nanorods were synthesized using electrochemical methods using polycarbonate membrane templates or porous alumina for shape control in the presence of surfactants (mostly CTAB) [34–35]. Because of their optical properties, gold nanorods became increasingly popular between 1999 and 2003 and a laborious three-step seed-mediated synthesis via a wet chemical route was developed [36]. The emergence of seed-mediated synthesis provided chemists, for the first time, with a versatile and convenient wet chemistry of synthesizing nanorods and several other anisotropic shapes such as rhombic dodecahedrons, tadpoles, cubes, and tetrapods [37–39].

Seed-mediated synthesis via wet chemical routes is undoubtedly the most promising and accepted method to synthesize anisotropic nanoparticles exhibiting superior plasmonic characteristics. The route allows chemists to control the reaction parameters for synthesizing nanomaterials of desired sizes and shapes. The method employs a growth solution consisting of the respective metal salt, a weak reducing agent, a structure-directing agent (predominantly quaternary ammonium surfactants) and silver ions for preferential facet binding in the solution phase. The seed-mediated approach is a multistep controlled redox reaction utilizing metal seed nanocrystals of 1.5–4.0 nm. The seed particles are synthesized by reducing precursor gold salt using an excess amount of a strong reducing agent, such as sodium borohydride (NaBH_4_). Although, seedless synthesis of anisotropic plasmonic metal nanoparticles has been reported, they involved binary surfactants for tuning the absorption spectrum [40].

Apart from CTAB, several other quaternary ammonium surfactants such as myristyltrimethylammonium bromide (MTAB), dodecyltrimethylammonium bromide (DTAB), hexadecyltrimethylammonium chloride (CTAC), and benzyldimethylhexadecylammonium chloride (BDAC) have been used as structure-directing agents for synthesizing gold nanorods [41–43]. Co-mixtures of these quaternary ammonium surfactants are also being used to synthesize anisotropic nanoparticles. Despite exhibiting such astonishing structure-directing features, surfactants are of limited use for the synthesis anisotropic nanoparticles for biomedical applications due to their cytotoxicity. The consequences, both in vitro and in vivo, are discussed in the following sections.

### Anisotropic nanoparticles: cytotoxicity of structure-directing agents

**Figure 2:**
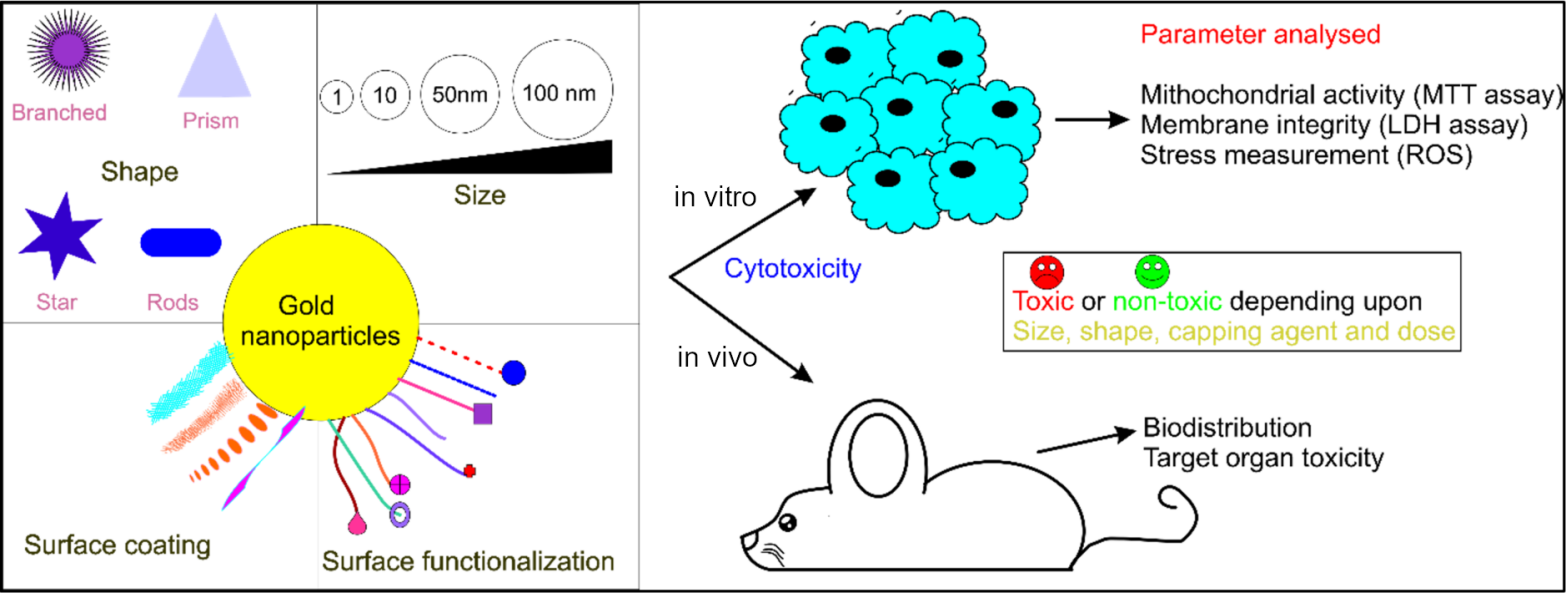
Cytotoxicity of anisotropic gold metal nanoparticles in vitro and in vivo.

The evolution of anisotropy through wet chemical methods (seed-mediated synthesis) using a surfactant (CTAB) has been discussed already. The function of surfactants as templates or matrices makes them an irreplaceable candidate for determining the controlled size/shape of anisotropic nanoparticles with excellent monodispersity. However, from the biological application point of view these nanomaterials could not achieve their potential despite acknowledgeable superior plasmonic properties. This is due to the toxic nature of surfactants adsorbed on the metal surface in a tightly packed bilayer structure. Among quaternary ammonium surfactants, CTAB has been extensively reported as cytotoxic even at low concentrations [44–46]. The positively charged CTAB interacts with the plasma membrane, which is negatively charged due to the asymmetric distribution of charged lipids between the two leaflets of the plasma membrane. This lead to a negative charged of the inner leaflet generating a surface potential and the binding affinity towards positively charged moieties. However, an interesting study revealed a structure-dependent cytotoxicity of quaternary ammonium surfactants in which cytotoxicity increased with the increase in carbon chain length of the surfactants [47]. Several reports have shown that the toxicity of CTAB-capped gold nanorods depends on nanoparticle size, shape, particle concentration, surface modification, and coating methods. There are contrasting results of toxicity based on various parameters conducted either in vitro or in vivo [48–53]. Gold is one of the most promising inert metals for synthesizing nanoparticles and is ideal for biomedical research. The safety and risk assessment of these nanoparticles are reported in the literature tabulated in Table 1. The cytotoxicity of nanoparticles is depicted based on several factors such as morphology, size and surface chemistry as shown in Table 1 and Figure 2.

**Table 1:** In vitro and in vivo toxicity studies of gold nanoparticles of different shapes with different surface groups.

Nanoparticles	Morphology	Surface group	Model system	Remarks	Ref.

gold	spheres	PEG	in vivo	acute toxicity to liver and spleen	[53]
gold	spheres	citrate	in vitro	dose-dependent cytotoxicity	[49]
gold	nanospheres, nanostars, and nanorods	chitosan	in vitro	toxicity trend: nanorods > nanostars > nanospheres	[54]
gold	nanorods	CTAB	in vitro	cytotoxic	[60]
gold	spheres	citrate	in vivo	size-dependent toxicity	[65]
gold	spheres, triangles, rods, trigonal bipyramids	CTAB	in vivo	genotoxic	[67]

### In vitro cytotoxicity of anisotropic nanoparticles

**Figure 3:**
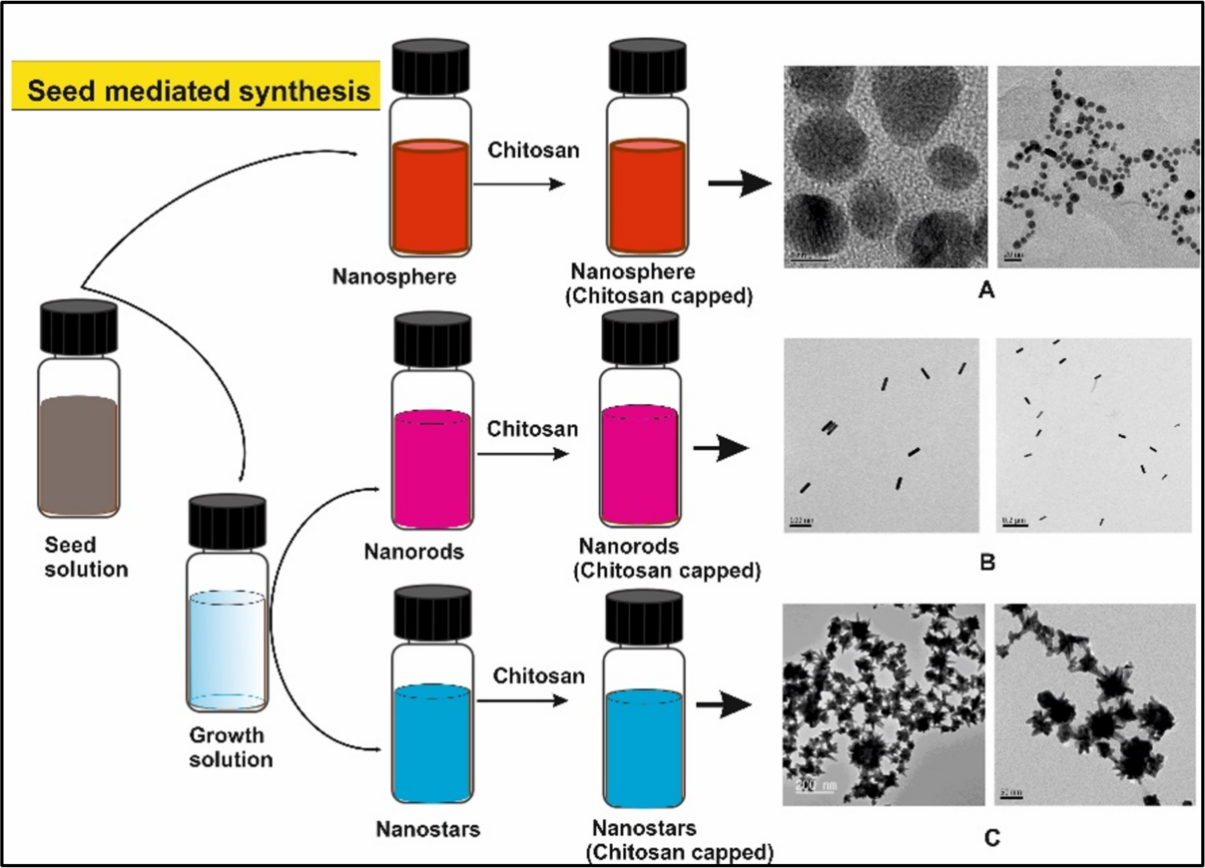
Synthesis of gold nanoparticles of different shapes using the seed-mediated approach. Chitosan was used for post functionalization of (A) gold nanospheres, (B) nanorods and (C) nanostars. Adapted from [54], © 2019 Y. J. Lee et al., distributed under the terms of the Creative Commons Attribution 4.0 International Licence, http://creativecommons.org/licenses/by/4.0/.

The use of anisotropic metal nanoparticles in biomedical research is gaining attention due to their plasmonic/optical properties. However, in vitro cytotoxicity assessment of these nanomaterials is a prerequisite for further in vivo validation and subsequent clinical trials. The physicochemical properties of a nanoparticle such as size, shape, and surface chemistry, determine their cytotoxicity. For example, gold nanoparticles of different shapes, as shown in Figure 3, displayed morphology-dependent cytotoxicity. Rod-shaped gold nanoparticles were more toxic than nanostars and nanospheres [54]. The potential risk is higher for anisotropic nanoparticles than for spherical shapes. This is due to greater exposed surface area and more defects during crystal growth of anisotropic nanoparticles. Gold nanoparticles show tremendous potential in biomedical research due to unique optical and physicochemical properties and the inert nanoparticle core. The inertness is due to metallic gold formed upon reduction of Au^3+^ during nanoparticle formation. However, there is also the possibility of Au^0^ oxidation that is influenced by size, shape, and stabilizing/capping agents. For instance, commonly used citrate and thiolate ligands result in a partial polarization of the nanoparticle core (Au^δ^ + O^δ^ and Au^δ^ + S^δ^), which cannot be neglected following its subsequent leaching [55]. It is also well known that gold cations play a key role in oxidizing substrates in aerobic redox reactions catalyzed by gold nanoparticles [56]. Redox reactions are intrinsic in biological organisms and are mainly governed by cytochrome P450, which acts as a strong catalyst for oxidation. Hence, the gold nanoparticles can alter the cell metabolism, leading to toxicity. In vitro studies also confirmed that isotropic gold nanoparticles with core sizes of greater than 5 nm were less toxic and considered biologically inert [44,57–58].

Numerous in vitro studies carried out using high-throughput techniques such as microscopic techniques, TEM, and ICP-MS revealed the fate of nanoparticles and their interaction at the interface between the metal surface and cell membrane. Electron microscopy revealed fundamental and mechanistic information regarding nanoparticle–cell interactions and their uptake into cell organelles. The metal content within the cells was determined via ICP-MS. Cell toxicity of nanoparticles depends on their size, shape, surface chemistry (predominantly determined by the capping agent used) and the cell type used for toxicity assessment. Chan and co-workers reported that cell internalization is optimum for nanoparticles with a size of 40–50 nm [59]. This is due to maximum interaction between antibody and receptor during receptor-mediated endocytosis. In vitro studies mostly involve a wide range of parameters, as highlighted by Murphy et al., but they do not provide all decisive aspects of toxicity [44]. John W. Stone and his group, in 2017, synthesized less toxic gold nanorods using dodecylethyldimethylammonium bromide (C_12_EDMAB) as an alternative structure-directing surfactant [60]. They carried out an in vitro cytotoxicity study exposing Hep-G2 and A549 cells to CTAB- and C_12_EDMAB-capped gold nanorods. The researchers observed a considerable difference in cell viability at the same concentration levels. Much earlier, a chemical method introduced by Chenxu Yu et al. illustrated the successful reduction of cytotoxicity of gold nanorods using organothiol compounds, namely 11-mercaptoundecaonic acid (MUDA) and 3-animo-5-mercapto-1,2,4-triazole (AMTAZ) [61]. PEGylation of gold nanorods is considered as a safe coating for alleviating the toxicity of CTAB adsorbed on the nanorod surface. The gold nanorods have excellent capability to be used in imaging as an optoacoustic contrast agent [62–63]. Poly(ethylene glycol) (PEG) is well known for reducing non-specific binding to biological molecules, rendering stealth character. This avoids macrophage recognition and phagocytosis and ultimately leads to prolonged blood circulation with enhanced retention and permeability of the nanorods. Apart from PEGylation, phosphatidylcholine has also been used as a coating agent for reducing the toxicity of CTAB-capped gold nanorods [64].

### In vivo toxicity of anisotropic nanoparticles

In vivo toxicity validation of nanomaterials is an inevitable step before clinical trials. However, in vivo studies of nanoparticles are subtle and quite controversial when compared to in vitro studies. The most common citrate-capped gold nanoparticles proved to be non-cytotoxic in vitro and showed size-dependent toxicity in vivo. Chen et al. revealed that gold nanoparticles of smaller (3–5 nm) and larger (30 and 100 nm) sizes were nontoxic in vivo; however, nanoparticles of average sizes 8, 12, 13, 37 nm were found to be toxic, provoking drastic weight loss, sickness, and short life span in mice [65]. The deteriorating effect was due to systemic toxicity evident through liver, lung, and spleen injury. In contrast, the non-cytotoxic nature of 13 nm citrate-capped gold nanoparticles has been reported [66]. Another recent study on *Drosophila melanogaster* showed mutagenicity of isotropic and anisotropic gold nanoparticles through the process of proton transfer (PT) [67]. A 1-(2-hydroxy-5-chlorophenyl)-3,5-dioxo-1*H*-imidazo[3,4-*b*]isoindole (ADCL)-based PT process on anisotropic gold nanoparticles was found to be accelerated compared to isotropic gold nanoparticles. The role of surface chemistry in determining toxicity and cell internalization of nanoparticles due to capping agents is critical. It has been reported that glutathione-capped gold nanoparticles underwent efficient cell internalization and efficient renal clearance compared to PEGylated and citrate-capped gold nanoparticles [68–69]. This was observed due to the difference in the binding affinity of the capping agents toward serum proteins. Glutathione showed less affinity towards serum proteins than citric acid. In an exposure time duration study by Lopez-Chaves et al., size-dependent metabolic fate and deposit formation of gold nanoparticles in different biological systems (liver, spleen, and kidney) of Wistar rats was observed [70]. The study revealed that gold nanoparticles of 10 nm size exhibited an oxidation-induced deleterious effect evident through nuclear localization and greater DNA damage. Despite oxidative imbalance induced by the gold nanoparticles, no inflammatory responses or tissue damage was observed for shorter exposure time. Therefore, the study concluded by commencing short time clinical use of these gold nanoparticles and understanding their use for chronic treatments better. A study was reported on the effect of CTAB-capped gold nanorods on estuarine model systems (consisting of sediments, plants, microbial films, fish, and snails) for observing ecological and environmental impact [71]. The results showed that the biofilms were the primary route through which gold nanorods enters the food chain.

It is very evident from the discussed in vitro and in vivo studies that the root cause of cytotoxicity is the structure-directing agent. Therefore, green chemistry has been implemented in synthesizing nanoparticles without compromising the characteristics of anisotropic nanoparticles intended for biological application. The following section of the review focuses on DESs and carrageenan, a class of emerging green solvents and a carbohydrate polymer for synthesizing safe plasmonic nanomaterials.

### Deep eutectic solvents in nanotechnology

**Figure 4:**
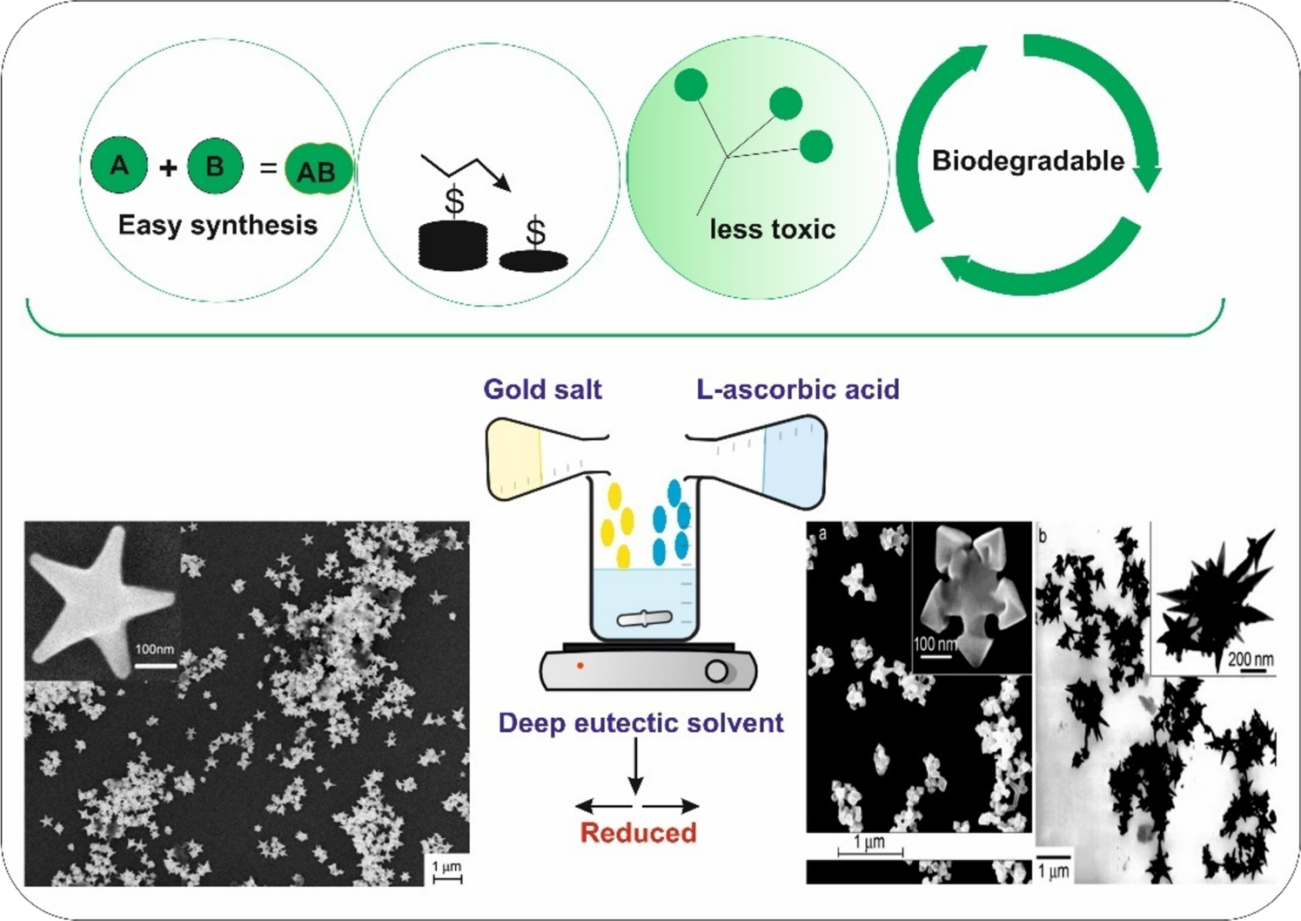
A deep eutectic solvent as green solvent in the synthesis of anisotropic nanoparticles (flower shape). All micrographs are adapted from [27], H. G. Liao et al., “Shape-Controlled Synthesis of Gold Nanoparticles in Deep Eutectic Solvents for Studies of Structure-Functionality Relationships in Electrocatalysis”, Angew. Chem. Int. Ed., with permission from John Wiley and Sons. Copyright © 2008 Wiley-VCH Verlag GmbH & Co. KGaA, Weinheim. This content is not subject to CC BY 4.0.

The preparation of DESs involves mixing solid organic precursors with high melting points, which interact via hydrogen bond to form a fluid at room temperature with a freezing temperature much below that of the individual precursor components. These strong hydrogen bonds restrict the recrystallization of the parent compounds [72]. There are numerous reports on DESs from various combinations of compounds by self-association between hydrogen bond donors and acceptors. The most extensively studied to date involve mixtures of choline chloride (hydrogen bond acceptor) with urea, ethylene glycol, or glycerol (hydrogen bond donors) in a molar ratio of 1:2 [25]. However, more DESs can be synthesized through selecting different components using permutation and combination. The wide range of components available allowed the chemists to fundamentally research the application of DESs. DESs are environmentally friendly, bio-degradable, and nontoxic, as shown in Figure 4. DESs were used as solvents for metal cleaning before the extensive use in electroplating. Electrolytic decomposition is another appealing process for developing a microscale propulsion system. Recent reports on the electrolytic decomposition of hydroxylammonium nitrate (HAN) were demonstrated [73–75]. DESs are well known for dissolving many species of high polarity, for example, amino acids, metal salts, glycerol, benzoic acid, citric acid, and glucose [26,76]. They are also promising in the dissolution of different polymers, such as starch, cellulose, lignin, chitin, and are also used in the pre-treatment of cellulose biomass [77]. Although the applications of DESs mentioned above lie at the interface of material and biological science, the following section will only discuss application of DESs in nanomaterial synthesis.

As mentioned earlier, the high solubility of metal salts in DESs makes them an ideal solvent. The solvation property determines the chemical (especially electrochemical) synthesis technology. Therefore, studying the solvation properties of a solvent is quite important for potential applications. DESs, due to their good solvation and electroconductivity properties, have been utilized in surface coating with nanoparticles through electrodeposition. A general electrodeposition setup consists of three electrodes, that is cathode, anode, and a reference electrode [78]. The solvation property and the conductivity of DESs also play a critical role in determining the physical structure, yield, and morphology of the products in chemical synthesis. This is also evident from the studies tabulated in Table 2, where the obtained shapes are given together with the eutectic mixture and precursor material used [79–87].

**Table 2:** Different DESs and their application in the synthesis of nanomaterials (NMs).

DESs	NM type	Surface group	Morphology	Role	Ref.

choline chloride/urea	alloys, iron	N/A, choline chloride-urea	dendrite-like and sharp-edged crystallites, nearly spherical	electrolyte for nanoparticle deposition, media for nanoparticle synthesis by a sputter deposition technique, solvent for chemical synthesis of nanomaterials	[79–80]
choline chloride/thiourea	chitin	acetic acid	whiskers	solvent for chemical synthesis of nanomaterials	[81]
choline chloride/1,3-dimethylurea	cobalt and nickel	N/A	coral-like	solvent for chemical synthesis of nanomaterials	[82]
choline chloride/malonic acid	cobalt	N/A	octahedral	solvent as well as structure-directing agent for chemical synthesis of nanomaterials	[83]
choline chloride/ethylene glycol	manganese	N/A	spherical	dispersant for nanoparticles and chemical synthesis of nanomaterials	[84]
choline chloride/acrylic acid	molybdenum, iron	poly(acrylic acid)	2D sheets	dispersant for nanoparticles and chemical synthesis of nanomaterials	[85]
choline chloride/*p*-toluenesulfonic acid	titanium	N/A	spherical	solvent as well as a structure-directing agent for chemical synthesis of nanomaterials	[86]
choline chloride/tris(hydroxymethyl)propane	graphite	epoxy resin	platelets	dispersant for nanomaterials	[87]

The aggregation/agglomeration of nanoparticles in the dispersion phase is a commonly encountered challenge. The stability of the nanoparticles is preserved by introducing various capping/stabilizing agents. The capping agent determines the surface chemistry of the nanoparticles deviating from the innate characteristics of the material. However, DESs when used as a solvent yield colloidally stable nanoparticles in the absence of capping/stabilizing agents. Also, the function of DESs is not limited to nanoparticle stabilization in their dispersion phase. They also act as a template, determining shape, size, and surface chemistry for the intended application. For example, Gutiérrez et al. synthesized porous carbon using *p*-toluenesulfonic acid and choline chloride in a molar ratio of 1:1 [88]. The DES used served as solvent and catalyst for the condensation of furfuryl alcohol, followed by carbonization resulting in the formation of pores. Oh et al. reported synthesizing highly monodispersed gold particles with a distinct rough surface and defined diameters using choline chloride and malonic acid [89]. The DES used served as reaction medium and structure-directing agent at the same time during synthesis. The synthesis did not require stabilizing or capping agents such as polymers or surfactants, highlighting the role of DES as a stabilizer and structure-directing agent. The work also illustrated temperature-dependent morphological differences between the nanostructures. Spherical sized small gold particles of nearly 100 nm were synthesized at 70 °C whereas network like nanostructures were observed when the synthesis temperature was 90 °C. Apart from nanomaterials, DESs are also being exploited for the electrodeposition of alloys for coating applications. For example, Bernasconi et al. developed a non-aqueous electrolyte using choline chloride and ethylene glycol in a molar ratio of 1:2 for electrodeposition of a zinc–nickel alloy to provide corrosion protection [90].

Due to the ever-rising interest in DESs for nanomaterial synthesis, a fundamental understanding regarding interfacial behavior and mass transport, such as ionic adsorption, surface wetting, double layered structure, and hydrogen bonding is needed as it will allow chemists to controllably manipulate the nanoscale growth [91]. While, in-depth studies (experimental and computational) regarding these aspects are yet to come, several significant preliminary studies have been reported. Much of the understanding of the interfacial behavior of DESs has been derived from electrodeposition studies. For example, Abbott et al. showed zinc electrodeposition in two different DESs (ethaline and reline in choline chloride), yielding respectively, “rice grains” and “platelets” morphologies [92]. The difference between the electrochemical double layers and a differential activity of chloride ions (i.e., preferential facet binding during crystal growth restricting the lattice growth in a particular direction) were responsible for the observed morphological difference. The lower surface tension of DESs facilitates rapid nucleation yielding tiny particles that undergo Ostwald ripening through a slow process, allowing for the manipulation on the nanoscale with controlled crystal growth.

### Plasmonic metal nanoparticle synthesis using DESs

Plasmonic metal nanoparticles, such as gold, silver, and platinum, are showing excellent potential owing to their unique physicochemical properties. DESs were first used for synthesizing anisotropic gold nanoparticles with high monodispersity in a mixture of choline chloride and urea in 2008 by Sun and co-workers [27]. The anisotropy was driven using the mild reducing agent ascorbic acid and the DES. The result was remarkable as the synthesis involved no surfactant or seeds. The Sun group also synthesized platinum nanoflowers of ca. 200 nm size with high monodispersity using DESs [93]. The group also successfully synthesized triambic icosahedral (TIH) Pt nanocrystals with high-index {771} facets. The nanoparticles with high-index planes exhibited higher catalytic activity due to high density of atoms with low coordination number compared to nanoparticles with low-index facets such as {100}, {111}, or {110}. Chirea et al. synthesized polycrystalline gold nanowires through a NaBH_4_-assisted rapid reduction of HAuCl_4_ in a DES (mixture of reline and ethaline) [94]. The strong coordination of [AuCl_4_]^−^ in reline exhibited a sixfold enhanced catalytic activity.

Apart from the high catalytic activity, nanoparticles produced in DESs showed no cytotoxicity in vitro systems. Guar-gum-fabricated gold nanoparticles (GA-GNPs) in a DES were synthesized for the use as X-ray contrast agent. The precursors for the DES used were choline chloride, gallic acid and glycerol. The X-ray attenuation coefficient of GA-GNPs was three times higher than that of the clinically used contrast agent Visipaque [95]. The in vitro study of the synthesized GA-GNPs confirmed their high potential to replace conventional contrast agents. In another example, Mahyari et al. synthesized gold nanoflowers using a DES without using reducing agents [95]. The gold nanoflowers showed excellent surface-enhanced Raman scattering (SERS) when doped with rhodamine B (RhB). The enhancement factor produced by these gold nanoflowers was estimated to be 1.09 × 10^5^ regarding pure RhB. The value of the enhancement factor is up to par with the intensively branched gold nanoparticles and is even greater than some of the reported gold nanostars and nanoflowers [96]. Concerning the biocompatibility of the nanomaterials synthesized using DESs, only a couple of studies has been carried out in vivo and in vitro. Mineral-substituted apatite nanoparticles synthesized using a choline chloride/thiourea mixture for prospective rejuvenation applications for bone tissues have shown no cytotoxicity in vivo [97]. The non-cytotoxic nature of fluorapatite nanoparticles synthesized using reline (choline chloride/urea) in vitro has been also reported [98]. However, with the rising popularity of DESs due to biodegradability, biocompatibility, sustainability, and low cost, achieving the real potential in nanobiotechnology is not far.

### Carrageenan as green/safe stabilizing agent in nanomaterial synthesis

**Figure 5:**
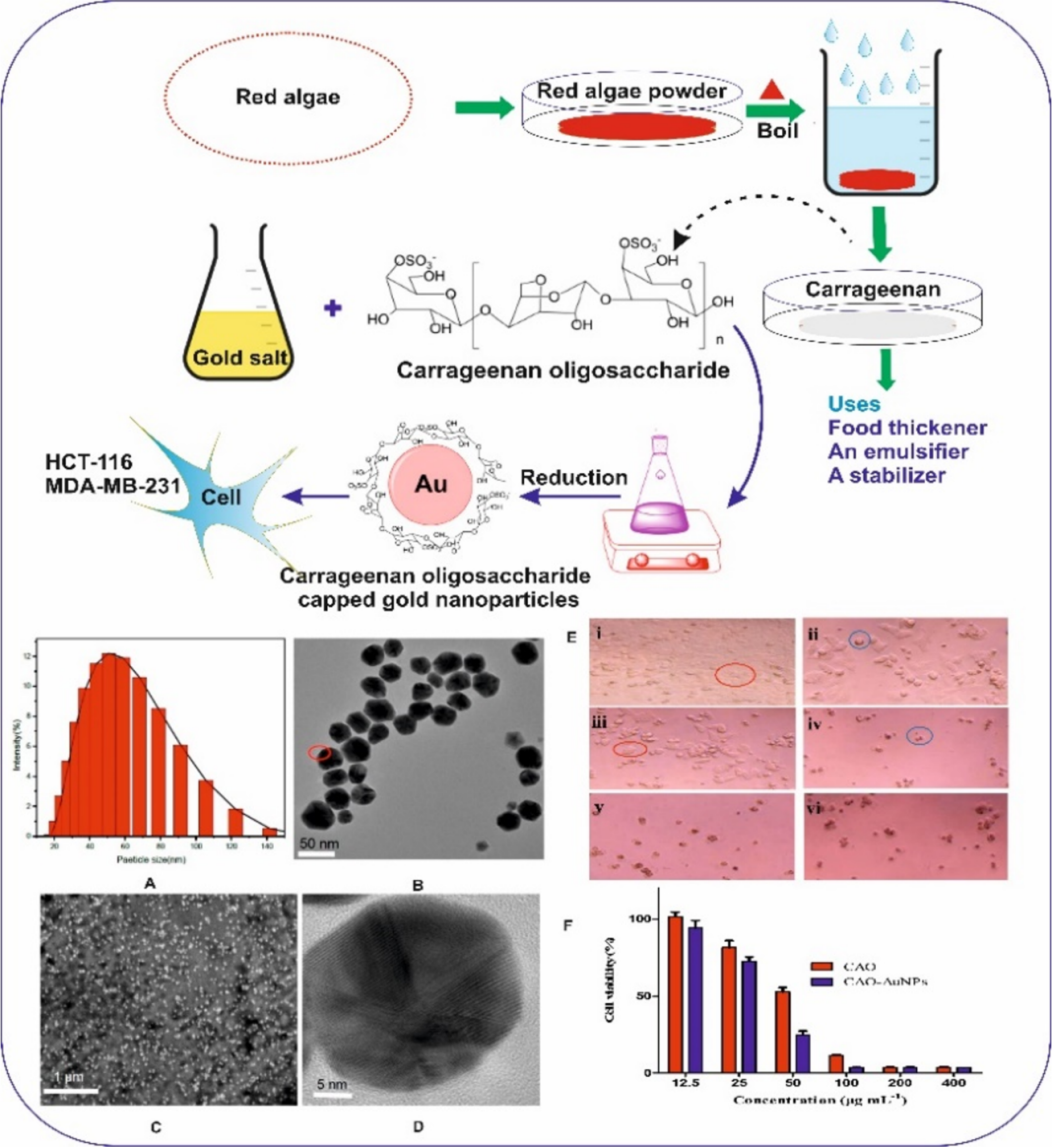
Carrageenan as capping and reducing agent for gold nanoparticle synthesis. (A) Histogram for the size distribution of nanoparticles. (B), (C) and (D) show TEM micrographs of the nanoparticles at different magnifications. (E) and (F) show the antitumor activity against MDA-MB-231 and HCT-116. Adapted from [32], © 2018 X. Chen et al., distributed under the terms of the Creative Commons Attribution 4.0 International Licence, http://creativecommons.org/licenses/by/4.0/.

A stabilizing agent, often known as capping agent, is one of the vital components in the synthesis of nanomaterials. The colloidal stability of the nanoparticles is governed by the capping agent, which prevents the aggregation of nanoparticles. Several capping agents such as CTAB, citrate, polymers, and carbohydrates are extensively used to stabilize nanoparticles in their colloidal state [99]. The capping agents govern the stabilization of nanoparticles and determine morphological changes in a nanoparticle due to differential binding to crystal facets during the crystal growth phase of the nanoparticle during synthesis. However, there are reports on synthesizing nanomaterials without stabilizing agents but they were very definitive in size-shape tuning and storage conditions [100–101]. Figure 5 shows the synthesis of gold nanoparticles using carrageenan as a capping/stabilizing as well as a reducing agent. Furthermore, these carrageenan-capped gold nanoparticles showed promising antitumor activity in MDA-MB-231 and HCT-116 cell lines [32].

Carrageenans are sulfated oligosaccharides extracted from red algae. They are composed of galactose and anhydrogalactose sub-units linked through a glycosidic bond. They are mainly categorized into three types depending on the degree of sulfation. The three types are: kappa carrageenan (one sulfate group per disaccharide), iota carrageenan (two sulfate groups per disaccharide) and lambda carrageenan (three sulfate groups per disaccharide). Carrageenan forms highly flexible curly helical structures, which are responsible for their gelation property at room temperature. The number of sulfate groups with repeating galactose units determines the gelation properties of carrageenan [102]. The higher the number of sulfate groups, the lower is the solubility temperature of carrageenan. Therefore, kappa and iota carrageenan do gelate whereas lambda carrageenan does not form gels at room temperature due to higher number of sulfate groups. All three carrageenans are soluble in hot water while lambda carrageenan is also soluble in cold water. Carrageenan is mainly used as an additive in the food industry for thickening, emulsifying, and preserving food and drinks [103]. Carrageenan is FDA-approved and remarkably safe [104]. In vitro and in vivo studies involving carrageenan proved that it is safe for biological applications with negligible inflammatory responses [105–107]. A conclusive study for evaluating cytotoxicity, intestinal permeability, and induction of proinflammatory cytokines of carrageenan was carried out using human intestinal cells (HCT-8 and HT-29) [108]. Also, carrageenan, due to the SO_3_^−^ groups, showed interaction with positively charged quaternary ammonium surfactants [109]. However, this sulfated oligosaccharide is yet to realize its full potential in the field of nanotechnology. The use carrageenan in nanomaterial synthesis and application has been tabulated in Table 3 [32,110–115].

**Table 3:** The use of carrageenan in synthesizing various nanomaterials (NMs) for potential chemical and biological applications.

NMs type	Surface group	Morphology	Role	Ref.

silica	κ-carrageenan	2-dimensional	wound healing	[110]
silver	κ-carrageenan	spherical	catalytic degradation of dyes	[111]
chitosan	alginate, carrageenan	spherical	drug delivery	[112]
iron	κ-carrageenan, ι-carrageenan, λ-carrageenan	spherical	self-assembled nanoreactor yielding magnetite nanoparticles with polymer encapsulation	[113]
silver	κ-carrageenan	spherical	food packaging	[114]
silver	κ-carrageenan	spherical	antibacterial activity and low cytotoxicity	[115]
gold	κ-carrageenan	spherical	antitumor activity	[32]

**Figure 6:**
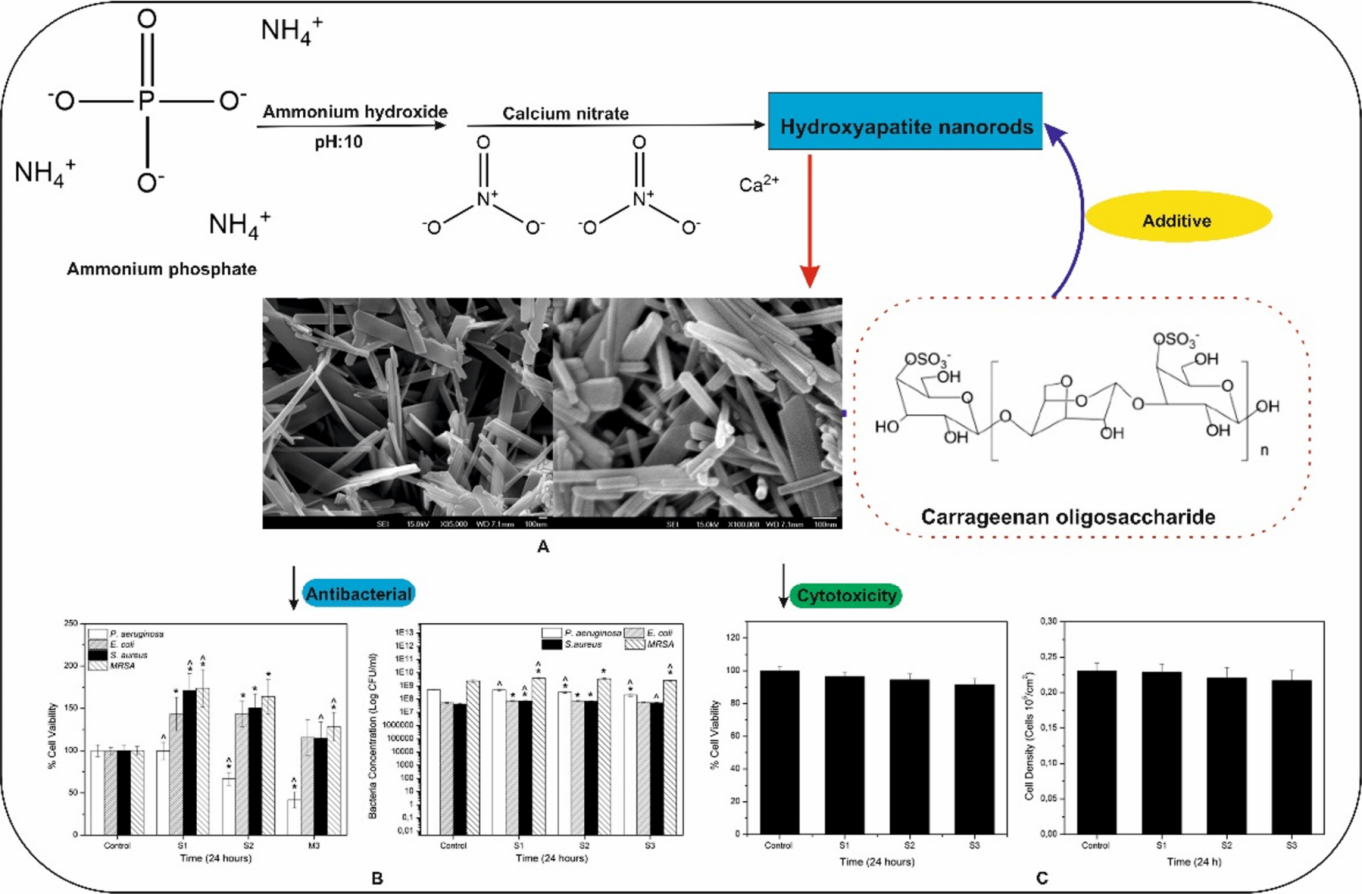
(A) A κ-carrageenan-stabilized hydroxyapatite rod-shaped nanocomposite. (B) Antibacterial study using *E. coli*, *S. aureus*, *B. subtilis*, *P. aeruginosa* showing the bactericidal properties of the nanocomposite. (C) The nanocomposite shows osteoblast cytotoxicity tests in cell lines (L02 and L929). Adapted from [116], J. I. González Ocampo et al., “Evaluation of cytotoxicity and antimicrobial activity of an injectable bone substitute of carrageenan and nano hydroxyapatite”, J. Biomed. Mater. Res. A., with permission from John Wiley and Sons. Copyright © 2018 Wiley Periodicals, Inc. This content is not subject to CC BY 4.0.

Carrageenan has been complexed with chitosan in a recent study, forming a composite for wound healing dressing. Silver nanoparticles, widely known for their antibacterial activity, are used in healthcare and the food industry, especially in manufacturing packaging materials. However, the cytotoxicity due to the release of the silver ions from AgNPs is a matter of concern. The cytotoxicity of micrometer-sized AgNPs was minimized by immobilizing them in carrageenan gel retaining the antibacterial property [85]. A study based on an injectable composite of carrageenan and nanoscale hydroxyapatite as an injectable bone substitute showed good adhesion properties with no cytotoxicity in vitro, as shown in Figure 6 [116]. The nanocomposite also exhibited antibacterial effect indicating its potential to restrict biofilm formation. Carrageenans are also being explored regarding the synthesis of polymeric nanoparticles complexed with other polymers such as chitosan and tripolyphosphate. The use of carrageenan is not only limited to biological applications, it also emerged as a promising candidate for industrial applications. Silver nanoparticles synthesized using carrageenan as a reducing and stabilizing agent showed promising results in removing organic dyes such as methylene blue and rhodamine B [111]. Magnetic iron nanoparticles were synthesized using κ-, ι-, or λ-carrageenan polysaccharides of different concentrations [113]. The particle size, morphology, and stability were predominantly determined by the concentration and the gelation properties of the used carrageenan. Another study involved stabilizing zein nanoparticles using ι-carrageenan to prevent aggregation and sedimentation above pH 5 [117]. The stability was enhanced upon adhesion of carrageenan to the nanoparticle surface rendering it negatively charged.

Although there are little reports on using carrageenan as a safe and sustainable component for synthesizing plasmonic metal nanomaterials, the works successfully illustrated the future endeavors for the oligosaccharide in the area of nanobiotechnology.

## Conclusion

This mini-review described two alternative vital components, DESs and carrageenan, in the wet chemical synthesis of plasmonic metal nanoparticles. Both components embrace the principle of green chemistry generating safe nanomaterials for biological applications. The authors also discussed the importance of CTAB as a structure-directing agent for anisotropic nanoparticles and the concerns regarding toxicity. One of the works that has been mentioned above involved C_12_EDMAB as an alternative to CTAB yielding less toxic nanorods. The lower toxicity can be attributed to the shorter carbon tail length of the surfactant compared to CTAB. Moreover, an excellent study reported the interaction between surfactants and carrageenan. The SO_3_^−^ group present in carrageenan interacts with the positively charged head group of the surfactants in the solution phase.

The importance of using DESs and carrageenan is the fact that the biocompatible molecules already used in designing safe nanomaterials add no further improvement than just biocompatibility. Some are also prone to enzymatic degradation. In contrast, DESs and carrageenan exhibit properties that play a role in enhancing the physicochemical properties of metallic nanomaterials. Intrinsic properties, such as structure-directing ability, were observed when DESs were used as solvents during nanoparticle synthesis. Also, the antibacterial, antiviral, and stabilizing properties of carrageenan can lead to a multidimensional approach in synthesizing nanomaterials for advanced biomedical applications.

A synthetic system comprising DESs and carrageenan along with surfactants of different carbon tail length can pave a route towards synthesizing plasmonic metal nanomaterials with controlled size and shape for biological applications. In this way, the presence of the surfactant shall cause minimal or no cytotoxicity while maintaining the integrity of the nanoparticles. The higher degree of solvation observed in DESs leads to the complete dissolution of carrageenan and surfactants. The dissolution of several compounds would significantly contribute to a species-rich system with higher conductivity. This unique hybrid model will create a platform for synthesizing *n* different nanomaterials with combinatorial possibilities of 2*^n^* since there are *n* possible ways of combining the components of DESs (hydrogen bridge donor and acceptor). Therefore, the authors believe that the combines use of the two components might allow for future endeavors towards synthesizing biocompatible plasmonic metal nanomaterials.
